# Implementing high value back pain care in private physiotherapy in Australia: A qualitative evaluation of physiotherapists who participated in an “implementation to innovation” system

**DOI:** 10.1080/24740527.2020.1732808

**Published:** 2020-05-18

**Authors:** Claire Gardner, G. Lorimer Moseley, Emma L. Karran, Louise K. Wiles, Peter Hibbert

**Affiliations:** a IIMPACT in Health, University of South Australia, Adelaide, South Australia, Australia; b College of Nursing and Health Sciences, Flinders University of South Australia, Adelaide, Australia; c Australian Institute of Health Innovation, Macquarie University, Sydney, New South Wales, Australia

**Keywords:** low back pain, physiotherapist perspectives, qualitative research, quality improvement, feasibility study

## Abstract

**Objectives**: Many barriers exist to delivering high-value care for people with low back pain (LBP). We have developed a multistrategy implementation system to overcome these barriers. Here we describe a qualitative evaluation of the experiences of private-sector physiotherapists implementing the system.

**Design**: PRISM (Practice-based innovation and implementation system) is an iterative clinician-as-scientist implementation program, tailored here for acute and subacute LBP. PRISM integrates strategies from behavioral change, implementation, and educational science fields. Semistructured interviews, group discussion forums, and electronic questionnaires were used to collect data at multiple time points that were then analyzed using an interpretative descriptive approach.

**Participants**: Six physiotherapists (purposive sample) practicing in private practice physiotherapy clinics in the Adelaide region, South Australia, were enrolled in the study.

**Interventions**: Interventions included an educational pain science and care workshop incorporating self-regulated learning principles, a co-planned clinical pathway, an electronic decision support tool, development and support of a community of practice, case study simulations, audit and feedback, and collaborative problem solving and innovation for physiotherapists.

**Results**: Participants’ experiences and perceptions centered around five themes: (1) knowledge and skills training; (2) networking and mentoring; (3) a clear clinical pathway; (4) practical tools; and (5) data feedback. Participants appraised the implementation process positively but identified patient receptiveness as a challenge at times. Suggestions for improvement included streamlining/automating data collection forms and processes and providing more simulation opportunities.

**Conclusions**: PRISM appears to be a promising approach to overcoming several barriers that prevent people with back pain from receiving high-value care. It consolidates and increases pain science knowledge and increases physiotherapist confidence in delivering high-value care. It appears to legitimize some current practices, enhance clinical reasoning and communication skills, extend knowledge in line with contemporary pain science, and facilitate the application of a biopsychosocial management approach. The high-level acceptance by participants provides a foundation for further research to test outcomes and delivery in different settings.

*Contribution of the article*

- A quality improvement intervention designed to improve delivery of high-value care was well received by private practice physiotherapists.
- Physiotherapists particularly valued using experiential learning to improve fluency in communicating with, and educating patients about, contemporary pain science.
- A structured clinical pathway and tools guided physiotherapists on the basic elements of necessary care and allowed them to concentrate on higher levels of decision making and communication with patients.

## RÉSUMÉ

**Objectifs**: Il existe de nombreux obstacles à la prestation de soins de grande valeur aux personnes souffrant de lombalgies. Nous avons développé un système de mise en œuvre multi-stratégies pour surmonter ces obstacles. Nous décrivons ici une évaluation qualitative de l'expérience des physiothérapeutes du secteur privé dans le cadre de ce système.

**Devis**: PRISM (Practice-based Innovation & Implementation System) est un programme itératif de mise en œuvre par les cliniciens en tant que scientifiques, adapté à la lombalgie aigüe et subaigüe. PRISM intègre des stratégies issues des domaines du changement de comportement, de la mise en œuvre et des sciences de l'éducation. Des entretiens semi-structurés, des forums de discussion de groupe et des questionnaires électroniques ont été utilisés pour collecter des données à plusieurs moments et analysés selon une approche descriptive interprétative.

**Contexte**: Cliniques de physiothérapie en cabinet privé.

**Participants**: Six physiothérapeutes (échantillon ciblé) de la région d'Adélaïde, en Australie méridionale.

**Interventions**: Un atelier de formation portant sur la science et les soins de la douleur intégrant des principes d'apprentissage autonome, un parcours clinique coplanifié, un outil électronique d'aide à la décision, l’établissement et le soutien d'une communauté de pratique, des simulations d'études de cas, l’évaluation et la rétroaction, ainsi que la résolution collaborative de problèmes et l'innovation pour les physiothérapeutes.

**Résultats**: Les expériences et les perceptions des participants se sont articulées autour de cinq thèmes : (1) l’acquisition de connaissances et de compétences ; (2) la mise en réseau et le mentorat ; (3) un parcours clinique clair ; (4) des outils pratiques ; et (5) la rétroaction. Les participants ont évalué positivement le processus de mise en œuvre, bien qu'ils aient déclaré que la réceptivité des patients constituait parfois un défi. Parmi les suggestions d'amélioration, citons la rationalisation et l’automatisation des formulaires et processus de collecte de données et l'augmentation des possibilités de simulation.

**Conclusions**: PRISM semble être une approche prometteuse pour surmonter plusieurs obstacles qui empêchent les personnes souffrant de maux de dos de recevoir des soins de haute valeur. Il consolide et augmente les connaissances en matière de science de la douleur et renforce la confiance des physiothérapeutes dans leur capacité de fournir des soins de grande valeur. Le programme a semblé légitimer certaines pratiques actuelles, améliorer le raisonnement clinique et les compétences en matière de communication, étendre les connaissances conformément à la science contemporaine de la douleur et faciliter l'application d'une approche de gestion biopsychosociale. Son haut niveau d’acceptation par les participants constitue une base pour la poursuite des recherches visant à tester les résultats et la prestation des soins dans différents contextes.

*Contribution de l’article*:

- Une intervention d'amélioration de la qualité conçue pour améliorer la prestation de soins de haute valeur a été bien accueillie par les physiothérapeutes en cabinet privé.
- Les physiothérapeutes ont particulièrement apprécié l'utilisation de l'apprentissage par l'expérience pour améliorer la fluidité de la communication avec les patients et les informer sur la science contemporaine de la douleur.
- Un cheminement clinique structuré accompagné d’outils a guidé les physiothérapeutes en ce qui concerne les éléments de base des soins nécessaires et leur ont permis de se concentrer sur les niveaux supérieurs de prise de décision et de communication avec les patients.

## Introduction

Low back pain (LBP) is the leading cause of years lived with disability worldwide.^1^ In Australia, LBP is the leading cause of early departure from the workforce^2^ and income poverty among older working-aged adults.^3^ The National Health Service in the United Kingdom recognizes LBP as a major contributor to sickness absence,^4^ and although current costs are unknown, they are almost certainly greater than the annual £10 billion estimated 20 years ago.^5^

LBP is a common yet complex disorder. Multiple physical, psychological, and social factors contribute to poor recovery and prolonged disability.^6,7^ LBP has a fluctuating course with often incomplete resolution.^8^ High-value care is defined as the best care for the patient, with the optimal result for the circumstances, delivered at the right price.^9^ Recommendations within evidence-based clinical guidelines define the specific features of high-value care for the management of patients with LBP and have existed for over 15 years,^10–13^ and there is broad consistency across the literature.^14,15^ Nonetheless, a substantial gap between evidence and practice still exists.^16,17^ For example, guidelines recommend that initial care should focus on exclusion of sinister pathology, education, reassurance, and graded return to activity, but only one in five Australian patients receive these.^17^ Though acute LBP is rarely associated with a clearly identifiable tissue lesion, the vast majority of patients are led toward unnecessary and potentially harmful investigations and pathology-focused care.^18^ The substantial evidence–practice gap results in the delivery of care that is considered low value, involving overtreatment or the provision of ineffective treatments. Such care has the potential to lead to unnecessary and preventable pain, disability, loss of productivity, extended delays (“sit and wait time”), and increased risk of developing chronic pain.^19^ It is patently clear that developing and passively disseminating guidelines and recommendations for high-value care per se do not change practice.^20^ Similarly, stand-alone education of health care providers in relation to recommendations generally has only a short-term impact.^21^

Physiotherapists are at the forefront of LBP management,^16^ yet they too frequently provide care that is considered low value.^22^ Two recent reviews on LBP show that many physiotherapists prefer to treat what they see as a biomechanical problem according to outdated structural–pathological models. They are reluctant to engage in the application of biopsychosocial recommendations in guidelines because they see evidence lacking applicability in the real world,^23^ and they tend to stigmatize patient behaviors that may suggest psychological or social aspects to their pain.^16,24^ Physiotherapists often perceive that neither their initial training nor professional development training provides the requisite knowledge base, skills, and confidence to successfully address and treat the complex and multidimensional nature of many LBP presentations.^24^

This already challenging situation may be further complicated by therapeutic drift. “Therapeutic drift” refers to the drop-off in skill levels of therapists over time; the variable use of empirically supported treatments, especially behavioral interventions; and the unsuitable implementation of such treatments. Therapeutic drift can potentially lead to further patient suffering and the public perception that treatments are ineffective.^25^ Self-perception among therapists does not align with therapeutic drift. That is, Walfish et al. found in a multidisciplinary practitioner survey that no therapists viewed themselves as performing below average, and 25% of the respondents said that they were in the top 10% of all therapists.^26^ Additionally, they rated the vast majority of their patients as improved, even though the best available data strongly suggest that this is unlikely to be true.^26^

That patients still often receive low-value care for LBP highlights the need for effective methods to change clinician behavior. Here we describe one such method, a quality improvement system called the Practice-based innovation and implementation system, or PRISM. PRISM integrates three synergistic best practice approaches into a single program: (1) contemporary educational strategies to optimize knowledge gain in biological sciences (in this case, pain science); (2) the best available implementation science methods from healthcare, and other industries such as education and aviation, to encourage the consistent delivery of high-value care for LBP; and (3) a clinical pathway that integrates essential components of high-value care from evidence-based guidelines and literature for LBP.

Here we report a qualitative evaluation of PRISM that aims to characterize the experiences and perceptions of the physiotherapists involved to inform the feasibility of sustainably integrating the PRISM intervention within private physiotherapy practice and the health care sector more broadly.

## Methods

We employed an interpretive descriptive qualitative study^27,28^ using a participatory approach to understand the experiences of physiotherapists in the private sector of implementing a high-value care system for back pain. Consistent with this approach, participants (physiotherapists) acted as active contributors, or clinicians-as-scientists, working collaboratively alongside researchers to shape the model of care and implementation of the intervention. A combined data collection strategy involved individual semistructured interviews (SSIs), group discussion forums (DFs), and an electronic questionnaire (EQ). A consolidated criteria for reporting qualitative research checklist for reporting qualitative studies is provided in Appendix A.^29^

### The Intervention—PRISM for Back Pain

**Figure 1.**
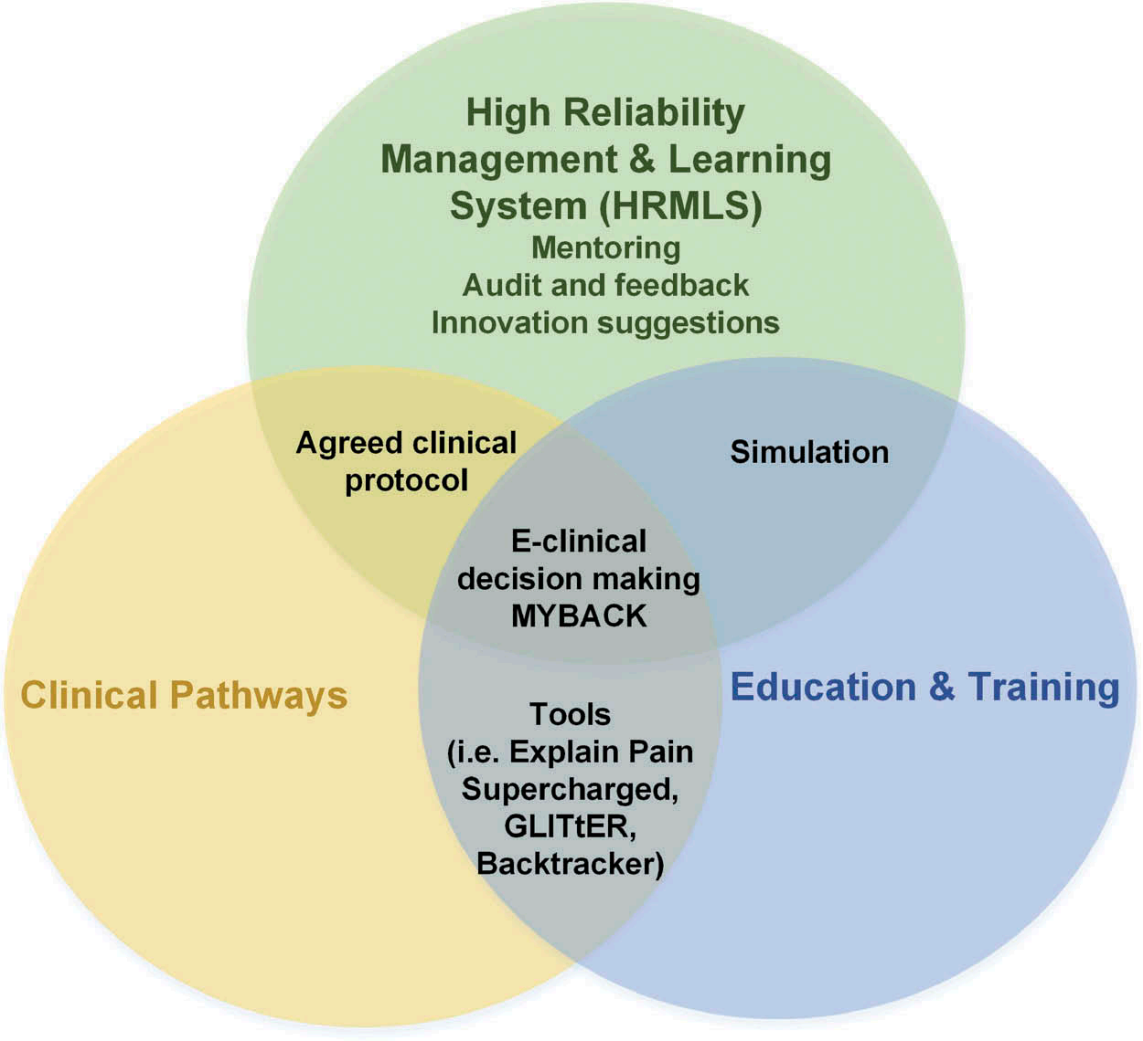
PRISM conceptual model

PRISM has three core components (Figure 1, Table 1). Here, the key participant in PRISM is a physiotherapist who takes the role of expert coach, providing the patient with high-quality information, reassurance, and pain education and facilitating informed self-management that is centered around graded physical activation according to a biopsychosocial model of pain. This contrasts with a traditional model in which the physiotherapist might take a role of pathology detector and corrector and/or pain reliever.

**Table 1.** Core components of PRISM

PRISM component	Description
1. Education and training	Contemporary educational strategies to optimize biological and clinical science pain knowledge and skills in pain care
2. Clinical pathways and collaborative innovation	Refinement and implementation of a high-value care patient pathway, including detection of sinister pathology, risk stratification, avoiding unnecessary imaging and medication (e.g., opioids), pain education, distress management training via brief cognitive behavioral therapy, refinement and implementation of clinical tools (see Table 2), and practical coaching for graded reactivation and self-management^17^
3. High reliability management and learning system	Establishing a community of practice, electronic clinical decision-making systems, an agreed-upon clinical protocol, audit and feedback, simulation and structured observation, mentoring and shared problem solving, data feedback, and suggestions on innovation

**Table 2.** Tools for the high-value care pathway

Tool	Features
Subjective patient questionnaire (Appendix B)	Patient questionnaire including data on patient demographics, back pain history, pain assessment, understanding of pain biology, catastrophizing, coping strategies, depression assessment, work status, recovery appraisal, and recovery expectations. Aggregated and de-identified comparative results from the questionnaire were fed back to participants
MyBack^31^	An electronic tool with an embedded algorithm to assist health professionals in understanding the level of risk associated with a patient’s signs and symptoms and triaging patients into management pathways based on risk
*Explain Pain*^32^	A patient resource providing content knowledge important for reassurance and pain science education. The book can be lent to patients to read through with carers/significant others
*Explain Pain Supercharged*^33^	A clinician’s guide for the content presented in the patient resource. Includes a guide to a pain assessment “cheat sheet”; planning conceptual change strategies; multiple examples of how to offer new concepts, engineer new experiences consistent with new concepts, and use resources that corroborate new concepts
GLITtER patient resources^34,35^	A 4-week patient guide that includes a framework for talking about common radiological findings in a manner that aims to reassure patients and promote activity
*The Explain Pain Handbook: Protectometer*^36^	A patient workbook integrated into care according to the cheat sheet findings
BackTracker	A bespoke tool to assist clinicians and patients to determine “normal” or “average” expected recovery rates. Can be used to guide reassurance and evidence-based escalation
Tamethebeast.org	An online resource that patients can read in their own time. It presents common pain-related target concepts and includes patient interviews in the form of podcasts
*Painful Yarns*^37^	A patient handbook that presents common target concepts

GLITtER = Green Light Imaging Interpretation to Enhance Recovery.

The PRISM principles that underline high value care are that there is often little or no relationship between back pain and demonstrable evidence of back injury; except in a small percentage of cases, imaging is unhelpful (i.e., not neutral), and the optimal pathway to recovery is a graded return to activity; modifiable cognitive and behavioral factors and social/work-related factors are the most powerful predictors of recovery; optimized reassurance and cognitive behavioral therapy–based coaching in self-management can modify these factors; and high levels of anxiety or depression are barriers to engagement in active rehabilitation and therefore to recovery, for which cognitive behavioral therapy is indicated.^15,30^

Participants attended three workshops consisting of a combination of participatory and self-reflective education and interactive simulated case scenarios. The aims of the workshops were to (1) provide foundational knowledge in pain science, (2) explain the PRISM intervention and implementation plan, and (3) introduce high reliability management and learning system and skills training to assist translation of knowledge and skills. Following the workshops, participants managed patients presenting with acute or subacute LBP in line with the PRISM clinical pathways. Discussion forums, which were teleconferences between the researchers and the participants, were held every 2 weeks for 6 weeks and then monthly for 3 months, followed by one forum 2 months later. The aim of the discussion forums was to support the participants and address any emerging problems associated with the PRISM model or the associated tools and provide an opportunity for participants to suggest improvements and ideas on innovation moving forward (innovation suggestions). Discussions at the workshops and forums were utilized as part of the study data collection (see Data Collection and Analysis). Integral to the PRISM approach is collaborative problem solving between researchers and clinicians, such that researchers are embedded in the implementation process and clinicians are embedded in the process of identifying and developing research ideas (clinician-as-scientist approach). In this way, PRISM can be considered an example of an implementation to innovation research–practice cycle.

Audit and feedback were achieved by collecting patient data by participants at three time points, initial consultation, 3-week follow-up, and 6-week follow-up, using the subjective patient questionnaire (see Appendix B). Appendix B Data were de-identified by participants and forwarded to the research team for analysis. Each participant’s patient data were aggregated, and participants were provided with a summary of all patient data and were able to compare their own patient data with those of the combined rest of the group but not with other individual participants.

### Study Design

We employed an interpretive descriptive qualitative study using a participatory approach.^27,28^ This approach is applicable where researchers have clinical knowledge of the subject matter, the researchers and participants co-construct understanding, and the output is designed to be practical for the relevant health professional group.^27,38,39^ It involves using a range of data sources and inductive and interactive analyses that are shared between the researchers and the participants to build understanding over time.^27,38,39^

### Sampling Strategy

A purposive sample of six physiotherapy practitioners was recruited. A small number were included because we aimed to gain a deep involvement with participants who were willing to share their experiences^27,38^ and to make our feasibility study logistically manageable.^40^ Eligible participants were registered physiotherapists in the Adelaide, South Australia, metropolitan region who are in private practice. Written and informed consent was obtained from each participant using project forms that included the names and professional qualifications of the research team. Participants were paid a AUS$500 honorarium in lieu of their time.

### Data Collection and Analysis

Data were collected between April 2018 and December 2018. Workshops were held in March, April, and June 2018 with telephone discussion forums commencing in May. Short debriefing sessions after each forum between the researchers allowed immersion in the data and theme development to occur iteratively and to inform subsequent discussions. Semistructured telephone interviews were conducted in July 2018, at approximately the halfway point of the study (Appendix C). Participants were sent the questions via e-mail prior to the interview. The nature of these interviews allowed for “at liberty” discussion and exploration of new ideas or thoughts by participants, consistent with the participatory approach of the study and focus on implementation to innovation. An electronic questionnaire (Appendix D) was circulated in December 2018 to complete data collection. In keeping with the interpretative descriptive design of the study,^27,28,38^ the questionnaire utilized open questions that were developed during the early analysis phase of the study and were informed by the emerging development of themes.

Two researchers (CG, PH) with experience in qualitative methods undertook the telephone interviews and discussion forums. One asked questions and the other took detailed notes, including verbatim quotations. The second researcher cross-checked notes. Interviews were not recorded due to participant preference, which was discussed in the workshops prior to data collection. The interviews, discussion forums, and electronic questionnaire underwent thematic analysis. Thematic analysis was inductive^41,42^ and focused on the semantic level.^41,43^ Analysis relied upon organizing sections of the data from all sources into recurrent themes with subthemes within these, allowing the data to suggest names for the themes and using direct quotations to illustrate the kind of data classified within each theme. This was an iterative process by one researcher (CG) to extract key themes and subthemes, which were then discussed with a second researcher (PH). One focus of the data analysis was themes related to informing the feasibility of PRISM. Emerging themes were shared between the research team and participants at workshops and discussion forums to ensure that interpretation of the themes were reflective of participants’ expression, to co-construct the narrative between researchers and participants, and to allow an agile and continuous relationship between data collection and analysis.^38,39^

## Results

Six physiotherapists (two females and four males) participated. Table 3 shows participant characteristics. Discussion forum length ranged between 30 and 45 min. Participation in the discussion forums was good, with an average of five participants in attendance across the forums. Follow-up one-on-one conversations were held when available for those who could not attend a given forum. Interviews were 30 to 60 min in duration and were conducted via phone. All participants completed the electronic questionnaire.

**Table 3.** Participant characteristics

Gender	Male	Female	Other		
	4	2			
Age (years)	< 30	31–40	41–50	50–60	61+
	1	3	2		
Years in profession	< 10	11–20	21–30	31–40	41+
	1	3	2		
Average number of acute LBP patients presenting per month	<5	6–10	11–15	15–20	21+
	3	2	1		

LBP = low back pain.

Evaluation of participants’ perceptions and experiences identified five core components that they believed to contribute to the impact of the PRISM intervention. These included knowledge and skills gains, networking and mentoring, a clear clinical pathway, practical tools and resources, and data feedback. In relation to the feasibility of PRISM, participants discussed the use of comparative data and their perceptions of patients’ receptiveness to such a model, and several suggested improvements to PRISM. These are summarized below and supported with verbatim quotes.

### Impact of the PRISM Intervention on Practitioner Development

Feedback from participants indicated that their involvement in the PRISM study was a positive experience and a valuable professional development opportunity

Being involved in PRISM has been a valuable experience and has reinforced best practice and provided flexibility with practical options for cognitive reassurance. (EQ, participant 2)

Overall PRISM has been a fantastic experience that I have been able to gain more experience and confidence in using the model and applying various tools in a clinic setting. (EQ, participant 5)

### Theme 1: Knowledge and Skills Gains

Although changes to knowledge and understanding of contemporary pain science were not formally tested, participants indicated that the training workshops helped to reaffirm and consolidate their understanding. This appears to have provided participants with a sense of confidence that their practice is consistent with evidence-based principles for managing LBP.

Hasn’t changed my understanding, however, has provided me with more information, greater access to tools enabling me to be more efficient and effective for clients. Has taken my practice to the next level. (SSI, participant 4)

Has provided more clinical reasoning regarding which pathway or education approach for individual patients. (SSI, participant 5)

[The changes] are mostly related to a lot more explaining and educating, keeping patients informed [by providing] “knowledge nuggets” and by explaining concepts aloud not just treating. A more collaborative approach and listening to patients. (SSI, participant 4)

Feels like it rolls off the tongue a little easier. (DF, participant 5)

Really enjoyed the simulation. Although found it confronting, the discussion and conversation around [the simulated scenario] was very beneficial. (DF, participant 4)

The workshop and simulated cases with the actor including the discussion following was very valuable. (SSI, participant 2)

A key change to practice was identified in how practitioners communicate with and educate patients and how they explain pain biology.

The simulated case scenarios coupled with access to tools and resources that focused on communication skills (Explain Pain Supercharged,^33^ Green Light Imaging Interpretation to Enhance Recovery [GLITtER]^34,35^) were particularly valuable for developing participants’ confidence and fluency when educating patients. The process of actively participating in the simulated case studies was beneficial; however, the opportunity to observe others and the discussion that followed was equally valued by participants. There was consensus that more simulated scenarios would have been beneficial, specifically practicing the use of the PRISM tools.

### Theme 2: Networking and Mentoring

Both the workshops and discussion forums provided participants with the opportunity to network and connect with “like-minded” peers.

I valued being a part of a group with like-minded practitioners, talking a similar language. It helped reinforce that I’m on the right track. Very valuable to hear from other practitioners, especially in private practice as you can get “stuck in your room,” so to speak, working in isolation. You can learn so much from talking with peers. (SSI, participant 3)

Provided a good opportunity to check in with others confirming using tools in similar ways/context and doing things the correct way. Practitioner input regarding things that are working well has been useful and has applied these learnings where relevant. They [discussion forums] have acted like an education opportunity somewhat. (SSI, participant 5)

Unique and awesome situation to have [expert] involvement. His comments have been very helpful. He captivates and educates and is a natural communicator. Highlights the value of a mentor or champion. (SSI, participant 3)

The discussion forums also appeared to serve as a mechanism to keep participants focused on the study and apply the PRISM principles in practice, retain accountability, and help minimize therapeutic drift. Similarly, the act of collecting patient data was highlighted as one of the mechanisms that encouraged participants to stay motivated and accountable to the PRISM principles and model of care. Having a content expert and well-respected leader in the field was also highlighted as an important component of the discussion forums and application of the project more broadly.

### Theme 3: A Clear Clinical Pathway

Participants indicated that the clinical pathway and systematic approach for managing patients with acute low back pain, including triggers for escalation (i.e., referral to a general practitioner for further investigations or for psychological support), was very helpful and integrating it into practice had been relatively easy.

The model and pathways were beneficial in terms of helping to de-threaten patients and were empowering for patients. The process also helped to reassure me regarding the treatment pathways. Provided me with confidence on how best to proceed with patients. (SSI, participant 4)

As physios we can sometimes over focus and overtreat. The PRISM model streamlines the process allowing for informed decision making. (SSI, participant 2)

Having very specific screening and clear pathways with escalation to CBT [cognitive behavioral therapy] and communication with GPs [general practitioners]. Have found the key target concepts to convey to patients very useful along with the language boxes. I really liked the “what to say” and “what not to say” when speaking with patients. (SSI, participant 1)

[The forms] were not super easy [to administer]. Patient presents in acute LBP and then asking them to do a questionnaire and educate about a study can be tricky to manage. Taking up time when patient wants pain relief. (DF, participant 3)

Benefits of the pathway were identified for both patient and practitioner. For example, the clear screening and triaging process at the beginning of consultations served to de-threaten and empower patients, reassure practitioners, and direct appropriate education right from the start.

A number of comments were made regarding the value of early collection and screening of psychosocial indicators, including depression, catastrophizing, recovery expectations, and the clear triggers and pathways for escalation. Key concerns from participants related to administrative requirements of the study in terms of educating patients about the study, obtaining consent, and completing relevant paperwork. A few participants found managing the administrative requirements of the study with patients who presented distressed with acute low back pain difficult at times.

### Theme 4: Practical Tools and Resources

The variety of user-friendly and practice-based tools provided to guide practitioner management of acute LBP was highlighted by participants as one of the key enablers for integrating the PRISM principles into practice.

The patient data questionnaire helped me determine the patients’ pain knowledge and thoughts about pain, allowing me to understand them quicker, determine the path of treatment faster, and provide more individualized education. (DF, participant 1)

My go-to resource (GLITtER). Provided something to hand out to patients (homework). Showed patients YouTube clips in consultation (to familiarize), patients responded very positively to these clips on return visits. (SSI, participant 5)

Imaging figure has been very useful. Was already using or quoting this data but didn’t have a tangible resource to show this. (SSI, participant 3)

Reassuring for acute patients that some pain at 2 weeks is normal. Useful in communicating and addressing expectations of patient’s and employer rework capacity in first few weeks. (DF, participant 6)

### Theme 5: Data Feedback

The patient data provided to participants was highly valued. The most clinically useful data included pain levels at initial presentation and follow-up, quality of life at initial presentation and follow-up, catastrophizing at initial presentation and follow-up, and active versus passive coping strategies at initial presentation and follow-up. The relationship between different data was highlighted as particularly interesting, specifically the data on understanding of pain biology and patient catastrophizing data.

Catastrophizing improves when you educate patients about what is normal, particularly around scans and imaging results. (DF, participant 6)

The data … will be valuable in speaking to and teaching my staff as it speaks to how quickly we can make changes to our client’s pain understanding if we take the time to educate properly. With what we know about how this can reduce threat; I think it will be valuable data to encourage my physios to begin this appropriate education early. (DF, participant 2)

Participants acknowledged that the data would be useful to demonstrate to patients who believe that they are “no better” that they are in fact improving and the areas where these changes are evident.

Participants did not perceive that patient data on mood were as important as the abovementioned data. Reasons given were that mood was not a primary objective or focus of physiotherapy treatment. Mood was acknowledged by participants as a good predictor of longer-term management and chronicity of pain, but they did not see mood management to be within the role of a physiotherapist.

#### Comparative Data

Participants supported receiving data that compared their patient data to group averages (all patient data). Because the participants’ data presented were predominantly consistent with group averages, this appeared to reassure participants about their practice.

I found the data reassuring that results for the most were similar between individual physios and the group average. (DF, participant 5)

It’s better to know so you can perhaps modify what you are doing. You can’t change something if you don’t know about it. (DF, participant 1)

Adds another dimension so you can see where you are in the pack. (DF, participant 2)

It was also noted that the comparative data were more meaningful than providing the group average data in isolation. This was supported even if personal data were not favorable or consistent with the group data. Participants expressed that this level of data feedback provided them with unique information and the opportunity to reflect on their practice.

### Participant’s Perception of Patient Receptiveness

The majority of participants indicated that the approach had been received well by their patients.

Patients generally excited by it—feels it makes practitioner sound a bit more like an expert being involved in the study. (SSI, participant 5)

If you go too strong with the active strategies too early. There is a real skill required to read and integrate concepts suitable for the individual patient. (SSI, participant 3)

The key is knowledge and training on how to implement this into treatment/therapy sessions. Many physio’s know about pain physiology etc. but are unable to implement this in an individual way that is specific to each patient, their beliefs and views. Access to easy to use infographics that outline evidence based care. (EQ, participant 3)

It was noted that the process of seeking patient consent to be involved helped to improve the credibility of the physiotherapist as an expert and may have also enhanced therapeutic alliance. One of the key challenges for participants, however, was managing patient expectations and beliefs while providing care in line with the principles of PRISM. Some participants indicated that some patients were less receptive to a pain education approach and expressed concern that these patients would seek care elsewhere. It was highlighted, however, that the training and tools provided as part of PRISM were valuable resources that helped address these challenges. Greater access to these resources was felt to be important for ongoing support of physiotherapists to provide high-value care.

### Suggested Improvements

Several suggested improvements were highlighted throughout the iterative process and especially within the electronic questionnaires. Time pressures and both the quantity and length of forms were the main barriers to using tools with patients. Suggested improvements included simplifying and streamlining data collection tools; further training that focused on how to use the tools in practice, including simulated examples; providing electronic versions of forms and tools; and setting up automated reminders to collect patient data.

Simplify data collection/outcome measurement tools. (EQ, participant 2)

A guide/info session earlier on in the sessions of how to use the tools would be useful to get a better understanding of how to integrate them into treatment, especially if they are tools that haven’t been used by the physio before. A role-play/example of someone using the tools in a clinical setting would accompany this well to give ideas/examples of how the tools can be used and applied. (EQ, participant 5)

A regular inbox reminder each week would continue to keep the collection of data front of mind and may result in more data collection as it’s easy to get caught up in the day-to-day grind and miss a back pain patient to include here and there. (EQ, participant 1)

Some participants suggested modifications to operational processes that proved helpful; for example, patients completing forms in waiting rooms.

## Discussion

We aimed to characterize the experiences of physiotherapists involved in PRISM Back Pain to inform the feasibility of sustainably integrating PRISM within private physiotherapy practice. The PRISM approach was well received by participants and viewed as a valuable professional development opportunity. Participants reported that the study both consolidated their knowledge and increased their confidence with managing patients who present with LBP. They felt that it legitimized their current practice and formalized and improved their clinical reasoning and professional judgment. The most notable change to practice was the reported improvements and fluency in communicating and educating patients about contemporary pain science and its application within a private physiotherapy practice context. The key factors that resonated well with participants were the structured and systematic approaches (clinical pathway, MyBack)^31^; access to, training in, and implementation of a range of practical tools (GLITtER,^34,35^ *Explain Pain*,^32^ and *Explain Pain Supercharged^33^*); and experiential learning (practical application of skills, simulations, and discussion forums). Our findings are consistent with literature on effective components of quality improvement interventions; for example, skills training via simulation and observation, data feedback on performance, mentoring and peer support, and the use of structured clinical pathways and tools.^20,44–47^

The nature of the therapeutic intervention in PRISM Back Pain is primarily education, reassurance, and guidance toward physical activation and consideration of psychological factors. As such, the physiotherapist uses his or her expert coach skills rather than focusing on a pathology detector and corrector skillset. This might be seen as a sensible progression because the validity of many structural pathology-based paradigms is either untested or has been refuted, which is partly why clinical guidelines emphasize education, reassurance, graded activation, and consideration of psychological factors.^48^ The expert coach approach seems to rely heavily on physiotherapists’ content knowledge and educational skills, as well as communication and behavioral change skills, broadly consistent with psychologically informed practice.^49^ These skills are not traditionally emphasized in undergraduate and continuing physiotherapy education,^50^ although, anecdotally, this is changing. A lack of training and high-level skills in these areas is one factor that probably underpins physiotherapists’ low confidence in adopting a comprehensive biopsychosocial approach when managing patients with LBP.^50^

The notion of psychologically informed physiotherapy brings with it an important consideration of scope of practice. That is, though physiotherapists might develop high-level skills in some psychological strategies, they are not psychologists. In the current study, participants reflected on the utility of tools and screening to facilitate timely and appropriate referral to psychological care. This is important because clinical pathways and associated tools have been criticized as being “cookbooks”^51^ that result in de-skilling health care providers, leaving them poorly equipped to contend with the variations between patients.^52^ However, our experience suggests that, when they are used optimally, they remind health care providers of the principles and basic elements of care; guide them in tailoring intervention to individual patients, including referral and progression; and allow them to concentrate on higher levels of decision making and interaction with patients. PRISM provided participants with a range of tools that appeared helpful but also preserved professional autonomy.^53,54^

### Next Steps

PRISM is an example of a complex quality improvement intervention grounded in contemporary sciences and based on evidence. It seems a feasible approach to improve care to patients with LBP.

One aspect of PRISM is that it is an iterative implementation approach: it adapts according to learnings for both participants and the research team. Broadening availability and access to both the tools and training were highlighted as important for future uptake, as were streamlining and automating data collection forms and processes and providing more simulation opportunities. PRISM could potentially be scaled up to a large number of practitioners by using blended models of information exchange; for example, via e-learning and face-to-face interaction. PRISM currently allows sufficient flexibility for individual practitioners and practices to adopt and implement within their respective business structures, which seems an important feature to preserve.

### Limitations

This study has several limitations. Our participants were motivated and opted into the study. Whether PRISM would have had as positive an impact with a less motivated cohort remains to be determined. All were private practice physiotherapists, so application of our findings is limited beyond that setting. Our sample was small; although this raises the possibility of missing key learnings we would have discovered with a larger cohort, it was intentional because we wanted to ensure feasibility of training and peer support and to collect qualitative data using a variety of means and at several time points. This study did not aim to determine the clinical efficacy of PRISM, which would be best served through alternative designs, such as randomized controlled trials.

## Conclusion

PRISM consolidated and expanded knowledge of contemporary pain science and its application within the private physiotherapy context. Participants felt that it boosted their confidence in their skills and practice, in recognizing when referral is indicated, and in implementing that referral process effectively and respectfully. Key aspects of PRISM were the content and skills training using simulation; peer and expert review and support; development of, training in, and feedback on the use of clinical tools such as GLITtER; feedback; and data comparison to group means. PRISM was perceived to be sufficiently structured and systematized to afford clear expectations of participants, yet flexible enough to be iterative and facilitate collaborative problem solving and innovation. A number of improvements could be made to PRISM, including automation and blended learning. The high level of acceptance by practitioners in the private physiotherapy setting provides a foundation for further research to test delivery and outcomes in different settings.

## Appendix A. Consolidated criteria for reporting qualitative research checklist.

**Appendix A.** Consolidated criteria for reporting qualitative research checklist

Item	Guide questions/description	Reported in section or page no.^a^
Domain 1: Research team and reﬂexivity		
Personal characteristics		
1. Interviewer/facilitator	Which author/s conducted the interview or focus group?	See Methods → Data collection and analysis
2. Credentials	What were the researcher’s credentials? For example, Ph.D., M.D.	See affiliations and titles
3. Occupation	What was the researcher’s occupation at the time of the study?	All researchers were researchers
4. Gender	Was the researcher male or female?	LM, PH: male; CM, EK, LW: female
5. Experience and training	What experience or training did the researcher have?	CG has experience in qualitative research methods that capture people’s perspectives on and experiences with a range of effects, including health professional training, evaluation of programs, and consumer input into and feedback on resources. She has actively engaged with developing interview guides, facilitating focus group and interview discussions with children and adults, thematic analysis and syntheses of qualitative data for reports, and, more recently, scientific publications. PH has undertaken semistructured interviews and focus groups with health care professionals, managers, and executives mainly in relation to patient safety and quality improvement over the last 15 years for the purposes of research and evaluation. He is the author of 14 peer-reviewed papers that undertook qualitative analysis of patient safety incident reports. EK has had relevant research experience in planning, conducting, and reporting feasibility studies of clinical interventions for low back pain and knee osteoarthritis. EK is also a registered physiotherapist. Over the last 7 years, LW’s primary qualitative research activities have centered around leading and analyzing data from focus groups and semistructured interviews with diverse stakeholder groups (e.g., health care professionals, researchers, policymakers, tertiary educators, consumers, and journal editors) to seek their perspectives on a range of topics. These include exploring their experiences of referral and triage processes for patients with spinal complaints, the use of clinical practice guidelines and their understanding of the underlying development processes, recommendations for defining roles and credentialing requirements for extended scope physiotherapy practitioners in Australia, the meaning of consumer engagement, the application of person-centered care principles in residential aged care, and journal publication practices and trends. In addition, Louise has coauthored several metasyntheses, which have facilitated skills in critical appraisal, analysis, and aggregation of data from primary qualitative studies. LW is also a registered physiotherapist. GLM is the professor of neuroscience and foundation chair in physiotherapy at the University of South Australia.
Relationship with participants		
6. Relationship established	Was a relationship established prior to study commencement?	There was no relationship between the study participants and the researchers prior to the commencement of the study.
7. Participant knowledge of the interviewer	What did the participants know about the researcher? For example, personal goals, reasons for doing the research	The participant information form that was provided with the consent form signed by the participant was explicit in its description of the purpose of the research and the professional qualifications of the researchers (CG, PH, LM). This information was provided to participants in more detail in the workshops.
8. Interviewer characteristics	What characteristics were reported about the interviewer/facilitator? For example, bias, assumptions, reasons, and interests in the research topic	One of the researchers (PH) was a facilitator who had worked in the same profession as the participants, meaning that he had shared experience of the clinical context in which the participants were applying their new knowledge. CG, who was not from the same profession, may have acted to mitigate bias from PH being from the same profession as the participants.
Domain 2: Study design		
Theoretical framework		
9. Methodological orientation and theory	What methodological orientation was stated to underpin the study? For example, grounded theory, discourse analysis, ethnography, phenomenology, content analysis	See Methods → Study design: Interpretive description
Participant selection		
10. Sampling	How were participants selected? For example, purposive, convenience, consecutive, snowball	See Methods → Sampling strategy
11. Method of approach	How were participants approached? For example, face-to-face, telephone, mail, e-mail	Participants were initially approached by e-mail and then a follow-up telephone when they were invited to participate in the study.
12. Sample size	How many participants were in the study?	See Methods → Sampling strategy: 6
13. Nonparticipation	How many people refused to participate or dropped out? Reasons?	No people refused to participate or dropped out
14. Setting of data collection	Where was the data collected? For example, home, clinic, workplace	See Methods → Data collection and analysis: Clinic, over the telephone.
15. Presence of nonparticipants	Was anyone else present besides the participants and researchers?	There was no one else present during data collection besides the participants and researchers.
16. Description of sample	What are the important characteristics of the sample? For example, demographic data, date	See Table 3
Data collection		
17. Interview guide	Were questions, prompts, and guides provided by the authors? Was it pilot tested?	See Methods → Data collection and analysis and Appendices C and D
18. Repeat interviews	Were repeat interviews carried out? If yes, how many?	Data were collected at multiple time points, including seven discussion forums, a semistructured interview, and an electronic questionnaire.
19. Audio/visual recording	Did the research use audio or visual recording to collect the data?	See Methods → Data collection and analysis
20. Field notes	Were ﬁeld notes made during and/or after the interview or focus group?	See Methods → Data collection and analysis
21. Duration	What was the duration of the interviews or focus group?	See Results
22. Data saturation	Was data saturation discussed?	Given the small number of participants, data saturation was not discussed.
23. Transcripts returned	Were transcripts returned to participants for comment and/or correction?	See Methods → Data collection and analysis
Domain 3: Analysis and ﬁndings		
Data analysis		
24. Number of data coders	How many data coders coded the data?	See Methods → Data collection and analysis
25. Description of the coding tree	Did authors provide a description of the coding tree?	Not applicable
26. Derivation of themes	Were themes identiﬁed in advance or derived from the data?	Themes were derived from the data (inductive)
27. Software	What software, if applicable, was used to manage the data?	Software was not used to manage the data
28. Participant checking	Did participants provide feedback on the ﬁndings?	See Methods → Data collection and analysis
Reporting		
29. Quotations presented	Were participant quotations presented to illustrate the themes/ﬁndings? Was each quotation identiﬁed? For example, participant number	Yes, see text
30. Data and ﬁndings consistent	Was there consistency between the data presented and the ﬁndings?	See themes in the results compared to the quotations in text
31. Clarity of major themes	Were major themes clearly presented in the ﬁndings?	See Results
32. Clarity of minor themes	Is there a description of diverse cases or discussion of minor themes?	No diverse cases (other than the spectrum of quotes presented in Results) or minor themes were discussed

^a^The third column either reports the section in the manuscript where the information is found or, if the information is not in the manuscript, a summary is provided.

## Appendix B. Subjective patient questionnaire

Physiotherapist code: _____________   Patient code: _____________

Date: _____________

**Patient details**

1. Gender:

□ Male   □ Female  □ Other

2. Age in years

□ Less or equal to 30  □ 31-40  □ 41-50    □ 51-60  □ Greater than 60

Back pain history:

3. How many weeks since the onset of back pain?

□ 0 to 2 weeks  □ 3 to 6 weeks   □ 7 to 12 weeks  □ 12+ weeks

4. How many episodes in the last 12 months have you suffered from back pain (including this episode)?

□ 1   □ 2    □ 3    □ 4   □ 5+

5. Any other health or orthopaedic issues?

_______________________________________________________________________________________________________

Pain Assessment

6. On average, how would you rate your pain over the last two days? (circle response)

0 = not painful at all     10 = excruciating pain

0  1 2 3 4 5 6 7 8 9 10

7. On average, how much has your back pain impacted on your quality of life over the last week? (circle response)

0 = not at all   10 = completely

0  1 2 3 4 5 6 7 8 9 10

8. Please answer true (T), false (F) or Unknown (U) to the following statements (some of these may be new terms or difficult to answer, just do your best and answer unsure where relevant).

**Table:**

		True	False	Unknown
1	It is possible to have pain and not know about it.			
2	When part of your body is injured, special pain receptors convey the pain message to your brain.			
3	Pain only occurs when you are injured or at risk of being injured.			
4	When you are injured, special receptors convey the danger message to your spinal cord.			
5	Special nerves in your spinal cord convey “danger” messages to your brain.			
6	Nerves adapt by increasing their resting level of excitement.			
7	Chronic pain means that an injury hasn’t healed properly.			
8	Worse injuries always result in worse pain			
9	Descending neurons are always inhibitory.			
10	Pain occurs whenever you are injured.			
11	When you injure yourself, the environment that you are in will not affect the amount of pain you experience, as long as the injury is exactly the same.			
12	The brain decides when you will experience pain.			

9. How often do you have these thoughts about your pain?

                     Never      Always

1. What if the pain doesn’t go away?      0 1 2 3 4 5 6 7 8 9 10

2. What if the pain spreads?         0 1 2 3 4 5 6 7 8 9 10

3. What if I can’t control the pain?         0 1 2 3 4 5 6 7 8 9 10

4. What if the pain becomes worse?        0 1 2 3 4 5 6 7 8 9 10

5. What if the pain is a sign of something worse?  0 1 2 3 4 5 6 7 8 9 10

6. What if the pain is so bad I can’t sleep?     0 1 2 3 4 5 6 7 8 9 10

7. What if I am unable to deal with the pain?    0 1 2 3 4 5 6 7 8 9 10

10. How do you currently cope with pain?

□ Passive (i.e. rest, taking pills – something is done TO you)

□ Active (i.e. movement or activity based strategies, learning about pain, seeking out SIMS – YOU do something)

11. How would you rate your average mood over the past week? (circle response)

0 = not at all depressed       10 = completely depressed

0  1  2  3  4  5  6  7  8  9  10

12. What is your current work status?

□ Full-time (normal duties)

□ Full-time (light duties)

□ Part-time (normal duties)

□ Part-time (light duties)

□ Not working

Recovery

13. Please rate your recovery (circle response):

0 = not at all recovered  10 = completely recovered

0   1   2   3   4  5  6  7  8  9  10

14. What are your expectations about recovery? (circle response)

0 = I don’t expect to recover at all    10 = I expect to make a full recovery

0   1   2   3   4   5   6   7   8   9   10

## Appendix C. Semistructured interview guide

1. What was the most valuable aspect of the PRISM workshops in supporting your understanding of the PRISM study and ability to implement the PRISM concept in practice?

2. How easy has it been to integrate the clinical model into practice (i.e., triage patients using MyBack and then following low- or med–high-risk pathways)?

3. What are the main barriers/enablers to integrating the PRISM model into practice?

4. Which of the PRISM tools has been most useful/utilized the most? Why?

5. Which of the PRISM tools has been least useful? Why?

6. What are the main barriers (if any) to using the tools with patients?

7. Overall what has been your patient’s response to the study?

8. How valuable have the physio discussion forums being? In what way? If not, why not?

9. Can you comment on the frequency of the discussion forums (i.e., fortnightly to monthly)

10. Has the PRISM study changed your understanding of pain science? If so, how?

11. Has the PRISM study changed the way you manage patients with LBP? If so, in what way?

12. If you could change or modify anything, what would it be?

13. Overall what has been the most valuable aspect of the PRISM study?

14. Is there anything missing or needed to better support you or other practitioners in the future?

## Appendix D. Electronic questionnaire

1. We are planning recruitment for PRISM 2 and would like to understand your reasons for participating in the current PRISM study. Please list below your motivations for joining PRISM.

2. What do you think are the core components of PRISM that should be included in future iterations?

3. What have been the key things that have kept you accountable to the PRISM principles and model of care?

4. Is there anything additional that would support you (or other physios) to provide high-value best practice care for low back pain?

5. Is there anything that we should change in the next version of PRISM” (provide details)

6. Would you be interested in being a mentor to other physios in PRISM 2?

7. Any other comments/feedback?

## References

[1] Vos T, Abajobir AA, Abate KH, Abbafati C, Abbas KM, Abd-Allah F, Abdulkader RS, Abdulle AM, Abebo TA, Abera SF, et al. Global, regional, and national incidence, prevalence, and years lived with disability for 328 diseases and injuries for 195 countries, 1990-2016: a systematic analysis for the Global Burden of Disease Study 2016. Lancet. 2017;390(10100):1211–59. doi:10.1016/S0140-6736(17)32154-2. 28919117 PMC5605509

[2] Schofield DJ, Shrestha RN, Passey ME, Earnest A, Fletcher SL. Chronic disease and labour force participation among older Australians. Med J Aust. 2008;189(8):447–50. doi:10.5694/mja2.2008.189.issue-8. 18928439

[3] Schofield DJ, Callander EJ, Shrestha RN, Percival R, Kelly SJ, Passey ME. Labor force participation and the influence of having back problems on income poverty in Australia. Spine (Phila Pa 1976). 2012;37(13):1156–63. doi:10.1097/BRS.0b013e31824481ee. 22166931

[4] National Health Service Employers. The back in work back pack: introduction and key messages. London (UK): NHSE; 2014.

[5] Maniadakis N, Gray A. The economic burden of back pain in the UK. Pain. 2000;84(1):95–103. doi:10.1016/S0304-3959(99)00187-6. 10601677

[6] Hartvigsen J, Hancock MJ, Kongsted A, Louw Q, Ferreira ML, Genevay S, Hoy D, Karppinen J, Pransky G, Sieper J, et al. What low back pain is and why we need to pay attention. Lancet. 2018;391(10137):2356–67. doi:10.1016/S0140-6736(18)30480-X. 29573870

[7] Australian Institute of Health and Welfare. Impacts of chronic back problems. Canberra (Australia): AIHW; 2016. Bulletin 137. Cat. no. AUS 204.

[8] Kent PM, Keating JL. The epidemiology of low back pain in primary care. Chiropr Osteopat. 2005;13:13. doi:10.1186/1746-1340-13-13. 16045795 PMC1208926

[9] Committee on the Learning Health Care System in America; Institute of Medicine. Best care at lower cost: the path to continuously learning health care in America. Vol. 8. Washington (DC): National Academies Press (US); 2013. 24901184

[10] National Institute for Health and Clinical Excellence. Early managment of persistent non-specific low back pain. Quick reference guide. London (UK): NICE; 2009.

[11] Delitto A, George SZ, Van Dillen L, Whitman JM, Sowa G, Shekelle P, Denninger TR, Godges JJ. Low back pain. J Orthop Sports Phys Ther. 2012;42(4):A1–57. doi:10.2519/jospt.2012.42.4.A1. PMC4893951 22466247

[12] National Health and Medical Research Council. Acute low back pain. Australian Acute Musculoskeletal Pain Guidelines Group. Evidence-based management of acute musculoskeletal pain. Brisbane (Australia): Australian Academic Press; 2003. p. 25–61.

[13] Rheumatology Expert Group. Therapeutic guidelines: rheumatology. Version 2. Melbourne (Australia): Therapeutic Guidelines Limited; 2010.

[14] O’Connell NE, Cook CE, Wand BM, Ward SP. Clinical guidelines for low back pain: a critical review of consensus and inconsistencies across three major guidelines. Best Pract Res Clin Rheumatol. 2016;30(6):968–80. doi:10.1016/j.berh.2017.05.001. 29103554

[15] Lin I, Wiles L, Waller R, Goucke R, Nagree Y, Gibberd M, Straker L, Maher CG, O’Sullivan PPB. What does best practice care for musculoskeletal pain look like? Eleven consistent recommendations from high-quality clinical practice guidelines: systematic review. Br J Sports Med. 2019;54(2):79–86. 10.1136/bjsports-2018-099878 30826805

[16] Gardner T, Refshauge K, Smith L, McAuley J, Hubscher M, Goodall S. Physiotherapists’ beliefs and attitudes influence clinical practice in chronic low back pain: a systematic review of quantitative and qualitative studies. J Physiother. 2017;63(3):132–43. doi:10.1016/j.jphys.2017.05.017. 28655562

[17] Williams CM, Maher CG, Hancock MJ, McAuley JH, McLachlan AJ, Britt H, Fahridin S, Harrison C, Latimer J. Low back pain and best practice care: a survey of general practice physicians. Arch Intern Med. 2010;170(3):271–77. doi:10.1001/archinternmed.2009.507. 20142573

[18] Maher C, Williams C, Chris L, Fellow R, Latimer J, Fellow S, Professor A. Managing low back pain in primary care. Aust Prescr. 2011;34:128–32. doi:10.18773/austprescr.2011.069.

[19] Mafi JN, McCarthy EP, Davis RB, Landon BE. Worsening trends in the management and treatment of back pain. JAMA Intern Med. 2013;173(17):1573–81. doi:10.1001/jamainternmed.2013.8992. 23896698 PMC4381435

[20] Flodgren G, Hall AM, Goulding L, Eccles MP, Grimshaw JM, Leng GC, Shepperd S. Tools developed and disseminated by guideline producers to promote the uptake of their guidelines. Cochrane Database Syst Rev. 2016;(8):Cd010669. 27546228 10.1002/14651858.CD010669.pub2 PMC10506131

[21] Scott SD, Albrecht L, O’Leary K, Ball GD, Hartling L, Hofmeyer A, Jones CA, Klassen TP, Kovacs Burns K, Newton AS, et al. Systematic review of knowledge translation strategies in the allied health professions. Implement Sci. 2012;7:70. doi:10.1186/1748-5908-7-70. 22831550 PMC3780719

[22] Traeger AC, Moynihan RN, Maher CG. Wise choices: making physiotherapy care more valuable. J Physiother. 2017;63(2):63–65. doi:10.1016/j.jphys.2017.02.003. 28325482

[23] Moseley GL. Let’s talk about us. Proceedings of the Australian Pain Society Annual Scientific Meeting: Gold Coast (Australia); 2019.

[24] Synnott A, O’Keeffe M, Bunzli S, Dankaerts W, O’Sullivan P, O’Sullivan K. Physiotherapists may stigmatise or feel unprepared to treat people with low back pain and psychosocial factors that influence recovery: a systematic review. J Physiother. 2015;61(2):68–76. doi:10.1016/j.jphys.2015.02.016. 25812929

[25] Waller G, Turner H. Therapist drift redux: why well-meaning clinicians fail to deliver evidence-based therapy, and how to get back on track. Behav Res Ther. 2016;77:129–37. doi:10.1016/j.brat.2015.12.005. 26752326

[26] Walfish S, McAlister B, O’Donnell P, Lambert MJ. An investigation of self-assessment bias in mental health providers. Psychol Rep. 2012;110(2):639–44. doi:10.2466/02.07.17.PR0.110.2.639-644. 22662416

[27] Thorne S, Kirkham SR, MacDonald-Emes J. Interpretive description: a noncategorical qualitative alternative for developing nursing knowledge. Res Nurs Health. 1997;20(2):169–77. doi:10.1002/(sici)1098-240x(199704)20:2<169::aid-nur9>3.0.co;2-i. 9100747

[28] Thorne SE. Interpretive description - qualitative research for applied practice. 2nd ed. New York (London): Routledge; 2016.

[29] Tong A, Sainsbury P, Craig J. Consolidated criteria for reporting qualitative research (COREQ): a 32-item checklist for interviews and focus groups. Int J Qual Health Care. 2007;19(6):349–57. doi:10.1093/intqhc/mzm042. 17872937

[30] Lim YZ, Chou L, Au RTM, Seneviwickrama KLMD, Cicuttini FM, Briggs AM, Sullivan K, Urquhart DM, Wluka AE. People with low back pain want clear, consistent and personalised information on prognosis, treatment options and self-management strategies: a systematic review. J Physiother. 2019;65(3):124–35. doi:10.1016/j.jphys.2019.05.010. 31227280

[31] Traeger AC, Henschke N, Hubscher M, Williams CM, Kamper SJ, Maher CG, Moseley GL, McAuley JH. Estimating the risk of chronic pain: development and validation of a prognostic model (PICKUP) for patients with acute low back pain. PLoS Med. 2016;13(5):e1002019. doi:10.1371/journal.pmed.1002019. 27187782 PMC4871494

[32] Butler DS, Moseley GL. Explain pain. Adelaide (Australia): Noigroup publications; 2013.

[33] Moseley GL, Butler DS. Explain pain supercharged. Adelaide (Australia): Noigroup publications; 2017.

[34] Karran EL, Yau YH, Hillier SL, Moseley GL. The reassuring potential of spinal imaging results: development and testing of a brief, psycho-education intervention for patients attending secondary care. Eur Spine J. 2018;27(1):101–08. doi:10.1007/s00586-017-5389-8. 29147798

[35] Karran EL, Hillier SL, Yau YH, McAuley JH, Moseley GL. A quasi-randomised, controlled, feasibility trial of GLITtER (Green Light Imaging Interpretation to Enhance Recovery)-a psychoeducational intervention for adults with low back pain attending secondary care. PeerJ. 2018;6:e4301. doi:10.7717/peerj.4301. 29404212 PMC5797685

[36] Moseley GL, Butler DS. The explain pain handbook: protectometer. Adelaide (Australia): Noigroup publications; 2016.

[37] Moseley GL. Painful yarns. Metaphors and stories to help understand the biology of pain. Adelaide (Australia): Dancing Giraffe Press; 2007.

[38] Teodoro IPP, Rebouças V, Thorne SE, Souza N, Brito L, Alencar AMPG. Interpretive description: a viable methodological approach for nursing research. J Escola Anna Nery. 2018;22(3):epub Mar 18.

[39] Hunt MR. Strengths and challenges in the use of interpretive description: reflections arising from a study of the moral experience of health professionals in humanitarian work. Qual Health Res. 2009;19(9):1284–92. doi:10.1177/1049732309344612. 19690208

[40] Bonney A, MacKinnon D, Barnett S, Mayne D, Dijkmans-Hadley B, Charlton K. A mixed-methods feasibility study of routinely weighing patients in general practice to aid weight management. Aust Fam Physician. 2017;46:928–33. 29464231

[41] Braun V, Clarke V. Using thematic analysis in psychology. Qual Res Psychol. 2006;3(2):77–101. doi:10.1191/1478088706qp063oa.

[42] Patton MQ. Qualitative evaluation and research methods. 2nd ed. Newbury Park (CA): Sage; 1009.

[43] Boyatzis RE. Transforming qualitative information: thematic analysis and code development. Newbury Park (CA): Sage; 1998.

[44] Scott I. What are the most effective strategies for improving quality and safety of health care? Intern Med J. 2009;39(6):389–400. doi:10.1111/j.1445-5994.2008.01798.x. 19580618

[45] Ivers N, Jamtvedt G, Flottorp S, Young JM, Odgaard-Jensen J, French SD, O’Brien MA, Johansen M, Grimshaw J, Oxman AD. Audit and feedback: effects on professional practice and healthcare outcomes. Cochrane Database Syst Rev. 2012;(6):Cd000259. 22696318 10.1002/14651858.CD000259.pub3 PMC11338587

[46] Ruiz-Gómez JL, Martín-Parra JI, González-Noriega M, Redondo-Figuero CG, Manuel-Palazuelos JC. Simulation as a surgical teaching model. Cir Esp (English Edition). 2018;96(1):12–17. doi:10.1016/j.cireng.2017.09.011. 29054573

[47] Registered Nurses’ Association of Ontario. Toolkit: implementation of best practice guidelines. Toronto (ON): Registered Nurses’ Association of Ontario; 2002.

[48] Foster NE, Anema JR, Cherkin D, Chou R, Cohen SP, Gross DP, Ferreira PH, Fritz JM, Koes BW, Peul W, et al. Prevention and treatment of low back pain: evidence, challenges, and promising directions. Lancet. 2018;391(10137):2368–83. doi:10.1016/S0140-6736(18)30489-6. 29573872

[49] Nicholas MK, George SZ. Psychologically informed interventions for low back pain: an update for physical therapists. Phys Ther. 2011;91(5):765–76. doi:10.2522/ptj.20100278. 21451090

[50] Cowell I, O’Sullivan P, O’Sullivan K, Poyton R, McGregor A, Murtagh G. The perspectives of physiotherapists on managing nonspecific low back pain following a training programme in cognitive functional therapy: a qualitative study. Musculoskeletal Care. 2019;17(1):79–90. doi:10.1002/msc.v17.1. 30468555

[51] Knaapen L. Evidence-based medicine or cookbook medicine? Addressing concerns over the standardization of care. Sociol Compass. 2014;8(6):823–36. doi:10.1111/soc4.12184.

[52] Timmermans S, Mauck A. The promises and pitfalls of evidence-based medicine. Health Aff (Millwood). 2005;24(1):18–28. doi:10.1377/hlthaff.24.1.18. 15647212

[53] Return to Work SA. The ‘Backtracker’ for health professionals. Adelaide (Australia): RTWSA: 2019.

[54] University of South Australia and FORM. Tame the Beast. Adelaide (Australia): University of South Australia; 2018 [accessed 2020 March 12]. https://www.tamethebeast.org/.

